# Bandwidth broadening of a linear polarization converter by near-field metasurface coupling

**DOI:** 10.1038/s41598-017-07296-4

**Published:** 2017-07-28

**Authors:** Xi Gao, Leena Singh, Wanli Yang, Jingjing Zheng, Haiou Li, Weili Zhang

**Affiliations:** 1 School of Information and Communication, Guilin University of Electronic Technology, Guilin, 541004 China; 2 School of Electrical and Computer Engineering, Oklahoma State University, Stillwater, Oklahoma 74078 USA; 3 Institute of Lightwave Technology, Beijing Jiaotong University, Beijing, 100044 China; 4 Guangxi Key Laboratory of Wireless Wideband Communication & Signal Processing, Guilin Guangxi, 541004 China

## Abstract

We experimentally demonstrate a highly efficient, broadband linear polarization converter functioning at terahertz frequencies. The linear polarization converter is composed of three metasurfaces and two dielectric layers interlaced with each other. The neighboring unit cells of the central metasurface layer of the linear polarization converter exhibit strong electromagnetic coupling, which increases the number of resonances and results in significant bandwidth broadening. The simulation and experimental results show that in the frequency range of 0.2 to 0.4 THz, the proposed polarization converter has a flat transmission curve and exhibits a transmission efficiency that is higher than 80%. High performance terahertz polarization conversion is desirable in many fields, such as terahertz spectroscopy, imaging, and communications.

## Introduction

The rapid advancement of terahertz science has led to the development of many practical applications in the fields of imaging, sensing, communications, and biomedicine^1–3^. For these applications, high-performance terahertz components, such as modulators^4, 5^, lenses^6^, wave plates^7^, switches, and polarization converters^8–10^, are essential for terahertz wave manipulation. Conventional polarization devices are generally realized by using birefringence in nematic liquid crystals or polymers^7, 11^ and are based on the mechanism of phase retardation between the two orthogonally polarized waves propagating along the device. Therefore, conventional polarization devices usually require a specific thickness and bulky configurations to obtain sufficient phase accumulation^12, 13^. It is extremely challenging to integrate these polarization converters within ultra-thin devices, such as advanced sensors and nano-photonic devices. Thus, novel approaches are desired to control the polarization state of the electromagnetic waves.

Metasurfaces, which are planar, two-dimensional, artificially engineered materials, have attracted extensive research interest for many potential applications^14–16^. By rationally designing the unit cells of the metasurfaces, one can achieve many exotic electromagnetic (EM) properties that have not yet been found in natural materials. Thus, metasurfaces provide a novel way to manipulate EM waves, including their polarization state. In recent years, many metasurface-based polarization devices with diverse functionalities, such as linear to linear, linear to circular, and circular to circular polarization conversion, have been demonstrated^17–19^. The polarization converters that function in transmission mode usually have the disadvantages of being narrowband and having a low polarization conversion efficiency. Hence, most of the high-performance devices operate mainly in reflective mode. Although a few novel metasurfaces have been proposed to improve the performance of the transmission polarization converter^20, 21^, achieving a high conversion efficiency over a broad bandwidth remains challenging.

In this letter, we present a linear polarization converter operating in transmission mode at terahertz frequencies. We experimentally and theoretically demonstrate that the proposed polarization converter is able to rotate linearly polarized EM waves by 90° in a broad bandwidth ranging from 0.2 to 0.4 THz and with an over 80% polarization conversion efficiency. Compared with other types of polarization converters^9, 21, 22^, the high conversion efficiency bandwidth of the proposed polarizer is effectively extended.

## Results

### Design and measurement of a double *L*-shaped metasurface-based polarization converter

**Figure 1:**
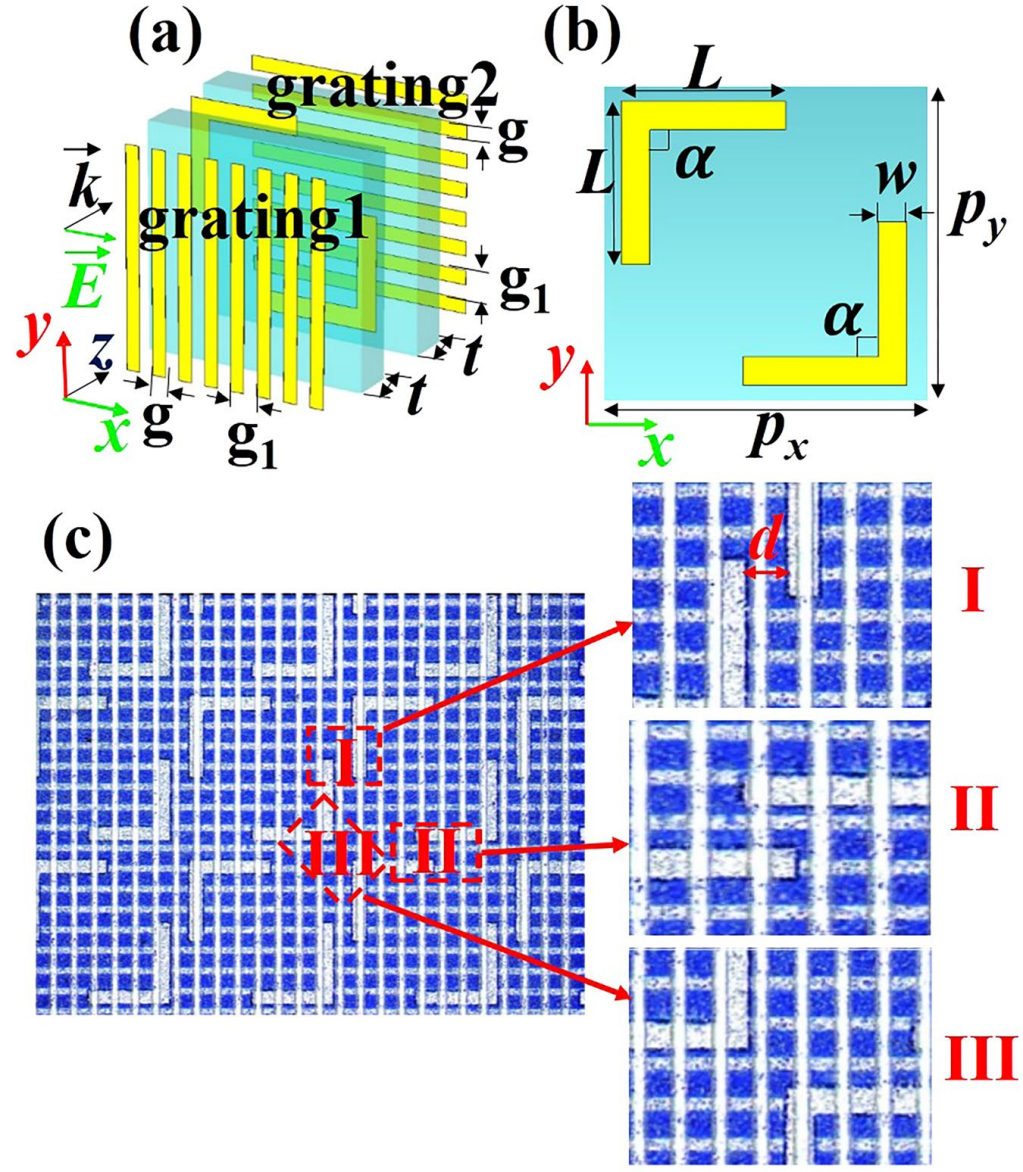
(**a**) Unit cell of the proposed polarization converter consisting of two gratings, a double *L*-shaped metasurface, and two dielectric plates. (**b**) Front view of the metasurface unit cell. (**c**) Optical image of the fabricated sample. The geometric parameters are *t* = 75 µm, *g* = 25 µm, *g*_1_ = 50 µm, *p*_*x*_ = *p*_*y*_ = 400 µm, *L* = 200 µm, *w* = 35 µm, and α = 90°. The periodic lattices are along the *x* and *y* directions, and the EM waves propagate along the *z* direction. The regions I, II and III, surrounded by the square frame, denote the EM mutual coupling areas of a unit cell with neighboring unit cells.

A schematic configuration of the proposed transmission-mode linear polarization converter is described in Fig. 1(a). It consists of two metallic gratings, a double *L*-shaped metasurface and two dielectric plates. Mylar (ε = 3.1 + 0.02*i*) films with thickness *t* = 75 µm are used as the dielectric plates, which are employed to separate the metallic gratings and the double *L*-shaped metasurface due to Mylar’s low dielectric losses. The two identical gratings with geometric parameters *g* = 25 µm and *g*_1_ = 50 µm are placed orthogonal to each other. Figure 1(b) shows the unit cell design of the middle layer metasurface, which consists of two *L*-shaped plasmonic antennas. The parameter α denotes the angle between the two arms of the *L*-shaped structure. Figure 1(c) illustrates the optical images of the fabricated sample, in which the light-colored parts are metal with a thickness of 0.2 µm. For any *L*-shaped structure, there are three regions (marked as regions I, II, and III in Fig. 1(c)) separated by only a short distance, *d*, where either the two arms or the corner angle of the *L*-shaped structure is closer to the counterpart of its neighboring *L*-shaped structure (see Fig. 1(c)). Because of this short distance *d*, significant EM mutual coupling usually occurs in these regions. In the following content (see Physical mechanism), we find that this EM mutual coupling in the three regions is the main contributor that leads to the performance enhancement of the proposed polarization converter. Furthermore, the distance *d* can be easily changed by altering the relative position of the two *L*-shaped structures, which makes it convenient to adjust the EM mutual coupling in regions I, II, and III to optimize the device performance.

**Figure 2:**
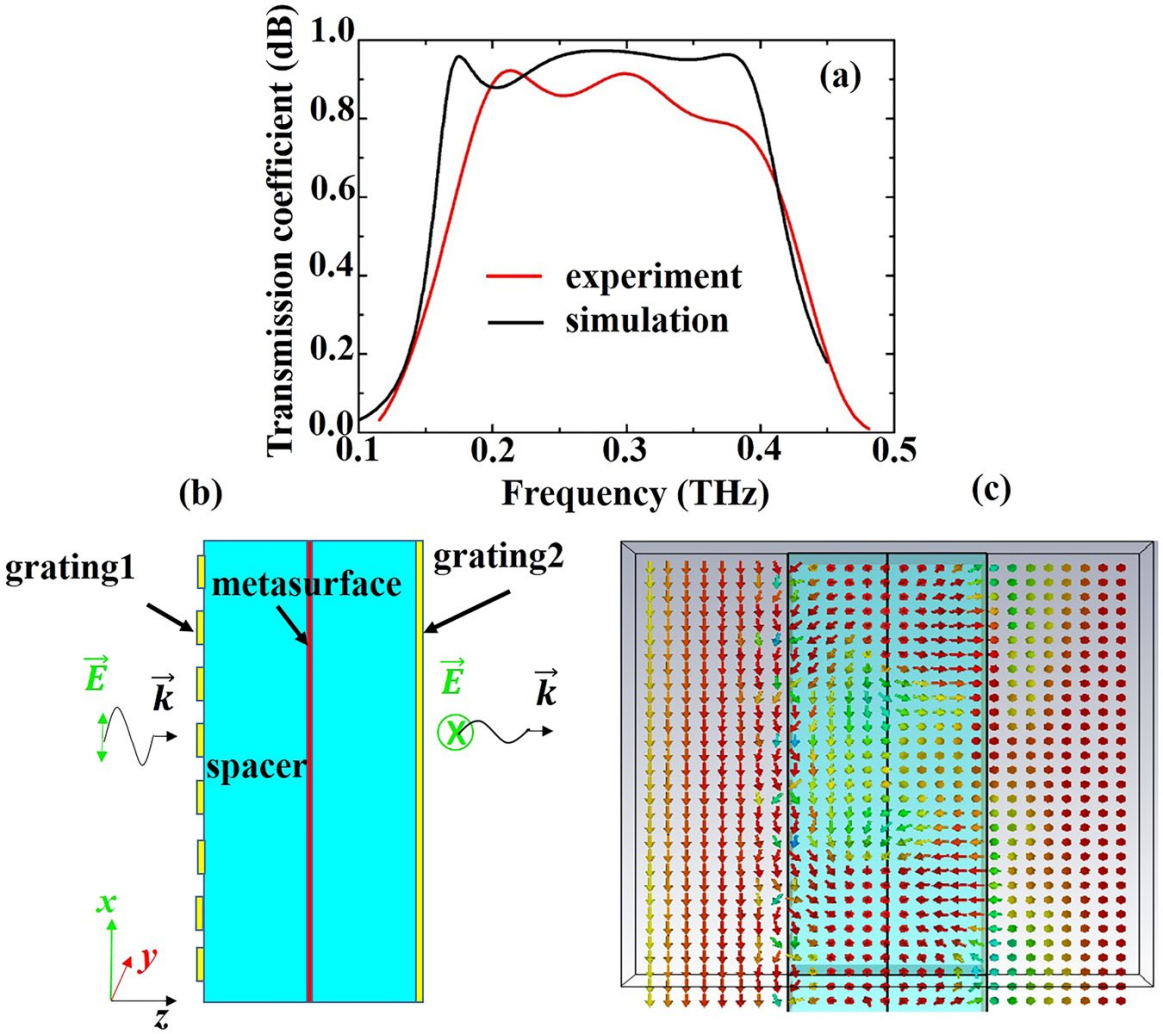
(**a**) Measured and simulated transmission spectra (*t*_*xy*_) of cross-polarization. (**b**) Cross section (in the *xz*-plane) of the proposed polarization converter. (**c**) Simulated electric field distribution in the cross section, which describes the polarization conversion process when the *x*-polarized EM wave propagates along the *z*-direction. The arrows denote the direction of the electric field.

To demonstrate the performance of the proposed polarization converter, we fabricated the sample and measured its transmission coefficient. Figure 1(c) shows the fabricated sample of the proposed device. We define *t*_*xy*_ = |*E*_*yt*_/*E*_*xi*_|^2^ and *r*_*xx*_ = |*E*_*xr*_/*E*_*xi*_|^2^ to denote the transmission and reflection coefficients. Here, *E*_*yt*_, *E*_*xi*_, and *E*_*xr*_ denote the electric fields of the *y*-polarized transmission wave, *x*-polarized incident wave, and *x*-polarized reflected wave, respectively. Figure 2(a) illustrates the amplitude of the cross-polarized (*y*-polarized) transmission coefficient for the *x*-polarized incident wave. It can be observed that the measured result agrees reasonably well with that of the simulation. In the experimental result, there is a frequency shift of approximately 0.02 THz, which may be caused by tolerances in the fabrication and measurement processes. From Fig. 2(a), we can also see that the proposed device shows a more than 88% transmission efficiency in the frequency range of 0.17 to 0.39 THz in the simulation. The measurement result reveals that the sample exhibits a more than 80% transmission efficiency in the broad bandwidth from 0.21 to 0.41 THz.

### Physical mechanism

**Figure 3:**
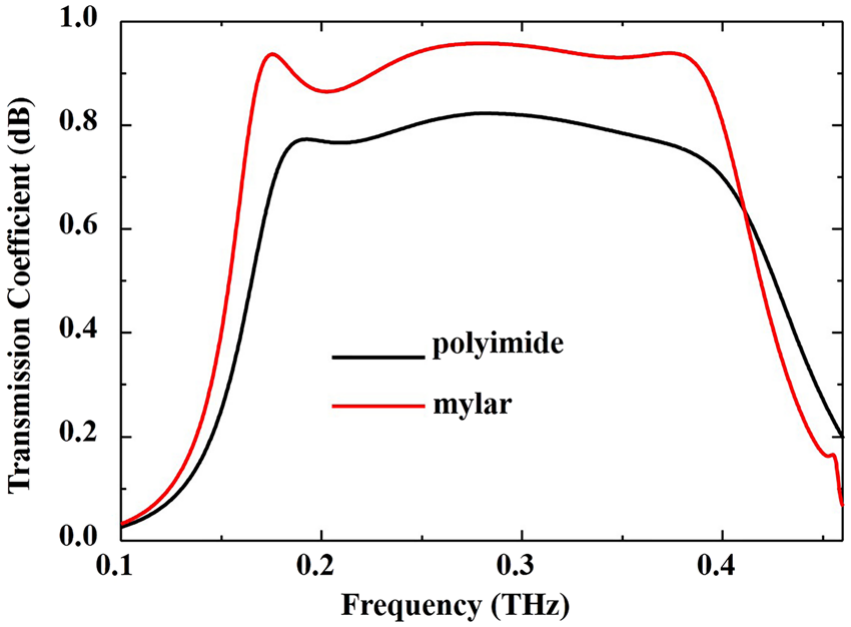
Transmission coefficient (*t*_*xy*_) for different dielectric spacers. The dielectric constants of polyimide and Mylar are 3.0 + 0.15*i* and 3.1 + 0.02*i*, respectively.

To elucidate the physical process of polarization conversion, the electric field distribution in the cross-section shown in Fig. 2(b) was simulated. Figure 2(c) illustrates the simulated result. When an *x*-polarized EM wave impinges onto the device from the +*z* direction, it first couples with grating 1 and is then rotated into a *y*-polarized EM wave by the metasurface layer. The rotated *y*-polarized EM wave then couples with the right side of grating 2. Consequently, the incident *x*-polarized wave is converted into a *y*-polarized wave when it passes through the polarization device. In this physical process, the dielectric losses due to the dielectric spacer and the electrical characteristics of the double *L*-structured metasurface directly impact the performance of the polarized device, including the bandwidth and the transmission efficiency of the proposed device. To demonstrate this, we investigated the polarization conversion of the proposed device with different losses in the dielectric spacer and different values of α (see Fig. 1(b)) by using the commercial software CST Microwave Studio. Figure 3 shows the comparison of cross-polarization transmission coefficient (*t*_*xy*_) for different dielectric spacers when other parameters are fixed. From Fig. 3, we can clearly see that the dielectric losses in the spacer can significantly affect the transmission efficiency of the device. When Mylar, with its low dielectric losses, is used as spacer, the transmission efficiency of the device is increased by approximately 15%. Therefore, we used Mylar as the dielectric spacer in our design.

**Figure 4:**
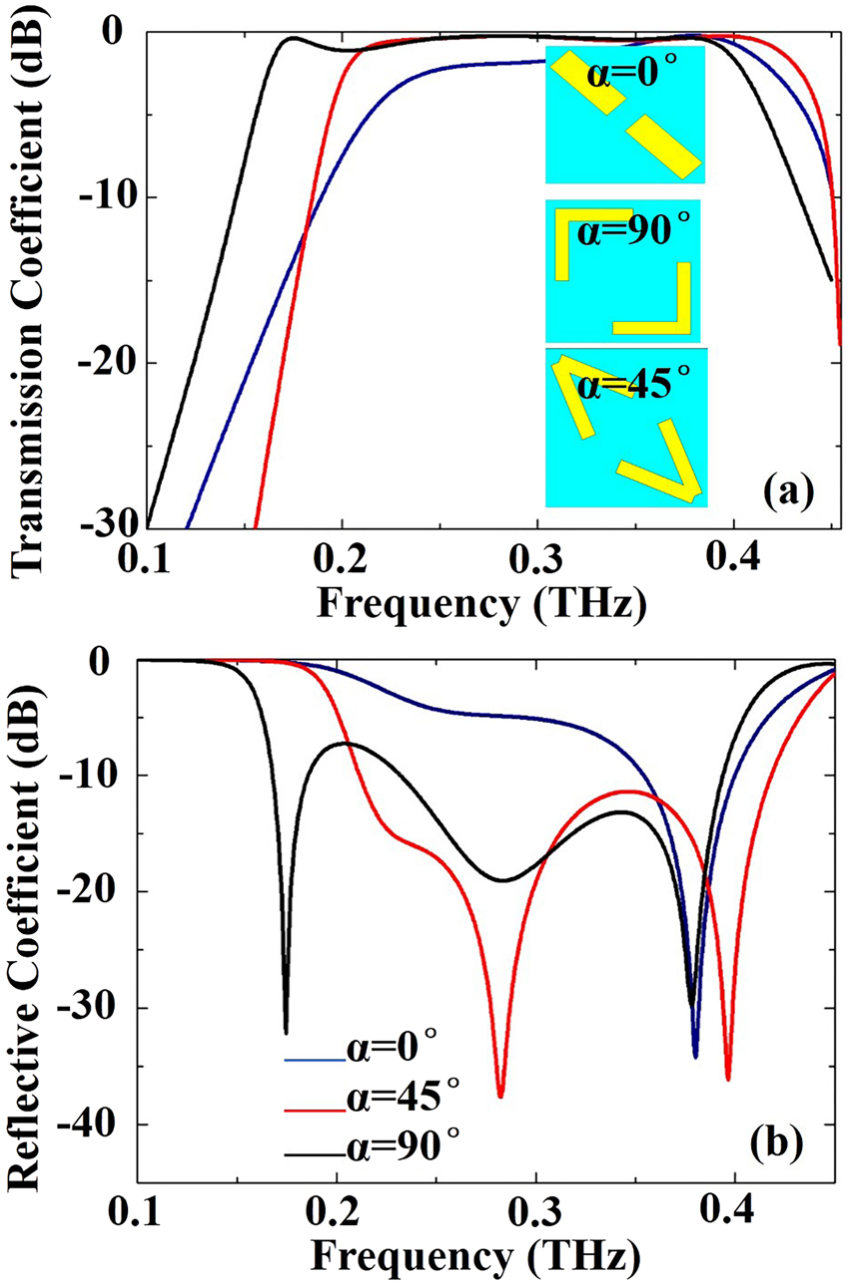
Simulated transmission (*t*_*xy*_) and reflection (*r*_*xx*_) coefficients for different α. The insets show the front view of the center metasurface layer for different α.

To investigate the performance of the device for different values of α, we first defined *Bf* = 2*(*f*_*h*_ − *f*_*l*_)/(*f*_*h*_ + *f*_*l*_) to denote the relative bandwidth, where *f*_*h*_ and *f*_*l*_ are, respectively, the upper and lower cutoff frequencies of the operation bandwidth. Figure 4 illustrates the transmission and the reflection coefficients for the three typical angles (α = 0°, 45°, and 90°). As shown in Fig. 4(a), the parameter α can effectively impact the bandwidth and the transmission efficiency of the polarization device. When α = 90°, the cross-polarization transmission coefficient (*t*_*xy*_) in the frequency range from 0.16 to 0.39 THz is greater than −1.1 dB, resulting in the relative bandwidth being 83.6%. When α is reduced to 45°, the relative bandwidth with *t*_*xy*_ over −1.1 dB is decreased to 66.6% (from 0.21 to 0.42 THz). Whereas when α is equal to 0°, the relative bandwidth is remarkably reduced to 16.2% (from 0.34 to 0.4 THz). Figure 4(b) illustrates the co-polarized reflection coefficients (*r*_*xx*_), from which we see that different α values correspond to different resonances. For α = 0°, the metasurface has only one resonance corresponding to *f* = 0.381 THz. When α increases to 45°, two resonance peaks appear at frequencies 0.282 and 0.396 THz. However, for α = 90°, three resonance peaks appear at 0.17, 0.28, and 0.37 THz, showing that the metasurface is a multi-resonance system when α is equal to 45° and 90°.

**Figure 5:**
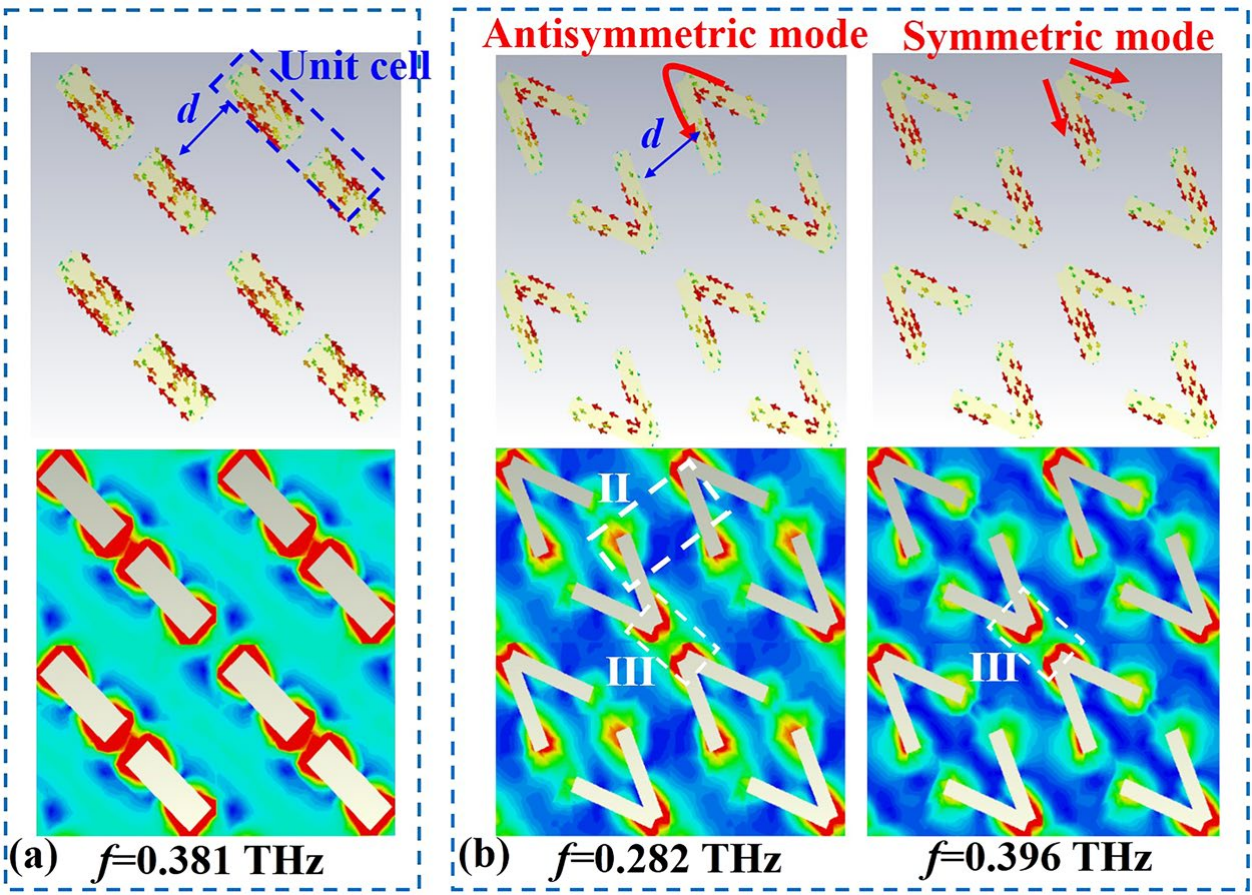
Surface current (upper parts) and electric field (lower parts) distributions on the middle metasurface; the red arrows denote the direction of surface current. (**a**) α = 0° and (**b**) α = 45°.

**Figure 6:**
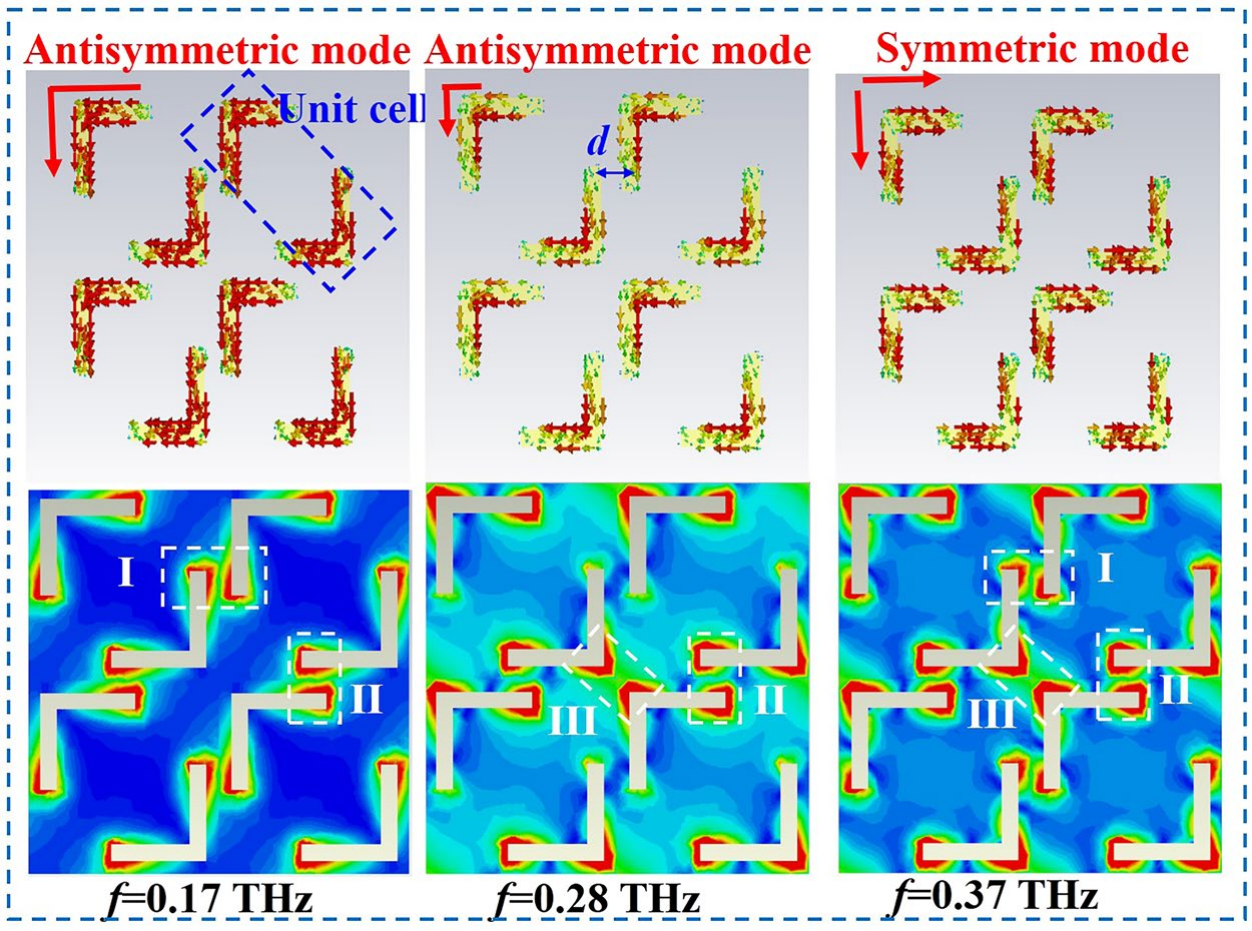
Surface current (the upper parts) and electric field distributions (the lower parts) on the metasurface layer with α = 90°.

To understand the physical mechanism of the multi-resonance system in further detail, we investigated the surface current and electric field distributions on the double *L*-shaped metasurface at α = 0°, 45° and 90°. For each value of α, we probed all the resonance frequencies, and the simulation results are shown in Figs 5 and 6. When α is equal to 0°, the double *L*-shaped structure is changed into a pair of cut wires, which is marked as a unit cell in Fig. 5(a). In this case, these cut wires can be considered equivalent to a LC resonant circuit that has only one resonance frequency at 0.381 THz. The surface current distribution shown in Fig. 5(a) verifies the LC resonance characteristic. On the other hand, we note that the distance (*d*) between the unit cell and its neighboring structure is the greatest, implying weak mutual coupling in this case. This decoupling characteristic can be further demonstrated by the electric field distribution (see the lower part in Fig. 5(a)), in which the largest electric field appears in only the gaps of the unit cell. When α is greater than 0° and less than 90°, for example, α = 45°, the metasurface acquires a double V-shaped structure, as shown in Fig. 5(b). According to reference^23^, the V-shaped plasmonic antenna can support two typical modes, the antisymmetric and symmetric modes. The current distributions for these modes are along the red arrows, as shown in Fig. 5(b). These different current distributions make the V-shaped metasurface resonate at two different frequencies (0.282 and 0.396 THz). Due to the existence of the two resonances, the bandwidth and cross-polarization efficiency for α = 45° are obviously improved. From the electric field distributions (see the lower parts in Fig. 5(b)), we see that at both resonances, the electric field in region II is very weak, which verifies the decoupling characteristic in this region. However, in region III, the electric field becomes strong, implying the enhancement of the EM mutual coupling.

For α = 90°, there are three resonances at 0.17, 0.28, and 0.37 THz, as shown in Fig. 4(b). We examined the surface current and electric field distributions at the three resonant frequencies as illustrated in Fig. 6. The unit cell is changed into a double *L*-shaped structure when α = 90°. In this case, the distance (*d*) is prominently reduced, which in turn leads to a significant increase in the mutual EM coupling. According to the surface current distributions (see the upper parts in Fig. 6), we deduce that the first two modes (0.17 and 0.28 THz) are symmetric modes and that the last mode (0.37 THz) is an antisymmetric mode. The two symmetric modes have similar surface current distributions but different electrical lengths. At the first resonance frequency (0.17 THz), the surface current is uniformly distributed along the two arms of the *L*-shaped structure, whereas at the second resonance frequency (0.28 THz), the surface current is mostly centered near the corner of the *L*-shaped structure, which corresponds to the reduction of the electrical length. Two resonances, which we attribute to the different electrical lengths, are formed. This physical phenomenon is caused by the EM mutual coupling between the adjacent unit cells. According to the electric field distributions (the lower parts in Fig. 6), we find there are three mutual coupling regions (regions I, II, and III). For the first resonance frequency, the strongest electric field appears in regions I and II, implying a strong mutual coupling in the two areas. However, for the second resonance frequency, the EM mutual coupling is switched to regions II and III. The different EM mutual coupling strengths in different regions cause the redistribution of the surface current, which leads to different electrical lengths and, consequently, different resonance frequencies. The symmetric mode, the metasurface with α = 90°, has a similar surface current as that with α = 45°.

Comparing the black line and the red line shown in Fig. 4(b), we observe that the resonance frequency of the symmetric mode for α = 90° is lower than that for α = 45°. This phenomenon can be explained in terms of EM mutual coupling. In the double *L*-shaped metasurface (α = 90°), there are three EM mutual coupling areas (regions I, II and III), whereas in the double V-shaped structure (α = 45°), only one region (III) shows strong mutual coupling. These results demonstrate that the multi-resonance characteristic of the double *L*-shaped metasurface results from the symmetric and antisymmetric modes, supported by the *L*-shaped antenna and the different EM mutual coupling between the neighboring unit cells, respectively. This multi-resonance characteristic plays a crucial role in significantly improving the performance of the proposed polarization device, including the bandwidth and the transmission efficiency enhancement.

**Figure 7:**
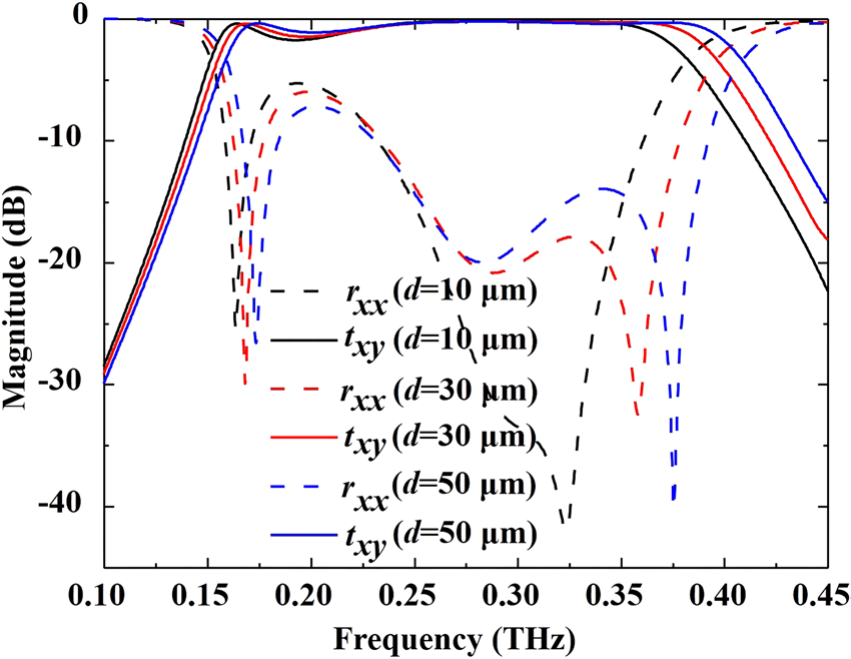
Simulated transmission (*t*_*xy*_) and reflection (*r*_*xx*_) coefficients for different *d* in the double *L*-shaped metasurface when other parameters are fixed. The solid lines correspond to *t*_*xy*_, and the dashed lines are *r*_*xx*._

For the double *L*-shaped metasurface, we also investigated the influence of parameter *d* on the performance of the proposed polarization converter. Here, we take three typical values of *d*, such as 10, 30 and 50 µm. Figure 7 shows the simulated transmission (*t*_*xy*_) and reflection (*r*_*xx*_) coefficients for the three *d* values. From the reflection coefficients, we see there are two resonances when *d* = 10 µm and that there are three resonances when *d* = 30 µm and 50 µm. Furthermore, these resonant frequencies show a blue shift when *d* is increased from 10 µm to 50 µm. The transmission coefficients also reveal that the device performance depends on the value of *d*. When the distance *d* is altered, the EM mutual coupling between the neighboring unit cells should be changed accordingly. The changed EM mutual coupling affects the resonance of the double *L*-shaped metasurface and further influences the performance of the proposed polarization device. So, the device performance can be optimized by choosing the appropriate value of the parameter *d*.

## Discussion

In conclusion, we have experimentally demonstrated that a metasurface consisting of double *L*-shaped plasmonic antennas can convert linear polarized waves into their cross-polarized waves over a broad bandwidth, ranging from 0.2 to 0.4 THz, with more than 80% transmission efficiency. We have also investigated the physical mechanism using numerical simulations. It is observed that by employing the *L*-shaped structure, the plasmonic antenna shows EM mutual coupling with neighboring antennas in different regions. Such mutual coupling behavior enables the metasurface to be a multi-resonance system and significantly affects the working bandwidth and transmission efficiency. By adjusting the mutual coupling reasonably, a polarization converter with good performance can be achieved. The demonstrated metasurface based polarization converter is an essential step toward high-performance integrated terahertz devices.

## Method

The polarization converter shown in Fig. 1(a) was fabricated with conventional photolithography and metallization processes. We start fabrication with two 75 µm thick sheets of Mylar. The double *L*-shaped metasurface and the grating are fabricated on the two sides of the first Mylar sheet; the other orthogonal grating is fabricated on the second Mylar sheet. Then, with the help of a microscope, we assemble the two structured Mylar sheets using the alignment shown in Fig. 1(a).

Figure 1(c) illustrates optical images of the fabricated sample, which contains 37 × 37 unit cells with an overall size of 15 × 15 mm^2^. The sample is experimentally characterized using broadband (0.1–4.5 THz) terahertz time-domain spectroscopy (THz-TDS). Specifically, the system consists of four parabolic mirrors placed between the transmitter and receiver, which are arranged in an 8-*f* confocal geometry enabling a frequency independent beam waist of 3.5 mm on the sample. An *x*-polarized (TE-polarized) light irradiates from the left side to excite the device (see Fig. 1(a)). On the other side of the sample, a polarizer is placed in a plane parallel to the *xy*-plane. The polarization direction of the polarizer is 45° with respect to the *x*-axis so that the cross-linear polarization transmission coefficient can be measured.
